# Pan-Cancer Analysis and Drug Formulation for GPR139 and GPR142

**DOI:** 10.3389/fphar.2020.521245

**Published:** 2021-02-19

**Authors:** Aman Chandra Kaushik, Aamir Mehmood, Xiaofeng Dai, Dong-Qing Wei

**Affiliations:** ^1^ Wuxi School of Medicine, Jiangnan University, Wuxi, China; ^2^ School of Life Sciences and Biotechnology, Shanghai Jiao Tong University, Shanghai, China; ^3^ Peng Cheng Laboratory, Vanke Cloud City Phase I, Guangdong, China

**Keywords:** GPR142, molecular modeling, pharmacophore, 3D quantitative structure-activity relationship, 7TM, pan-cancer, the cancer genome atlas

## Abstract

GPR (G protein receptor) 139 and 142 are novel foundling GPCRs (G protein-coupled receptors) in the class “A” of the GPCRs family and are suitable targets for various biological conditions. To engage these targets, validated pharmacophores and 3D QSAR (Quantitative structure-activity relationship) models are widely used because of their direct fingerprinting capability of the target and an overall accuracy. The current work initially analyzes GPR139 and GPR142 for its genomic alteration via tumor samples. Next to that, the pharmacophore is developed to scan the 3D database for such compounds that can lead to potential agonists. As a result, several compounds have been considered, showing satisfactory performance and a strong association with the target. Additionally, it is gripping to know that the obtained compounds were observed to be responsible for triggering pan-cancer. This suggests the possible role of novel GPR139 and GPR142 as the substances for initiating a physiological response to handle the condition incurred as a result of cancer.

## Introduction

Computational biology and immunoinformatic are the multidisciplinary fields that are progressing rapidly (Wadood et al., 2017). Besides, *in vitro*, and *in vivo* techniques are extremely demanding but still relatively challenging because of various factors such as resources, time of the experiment, experimental labor, cost, and environmental issues and safety as compared to the computational approaches. However, combining both these *in silico* and *in vitro*/*in vivo* techniques is highly essential for addressing a particular biological condition or targets such as the decryption of an immune response and vaccine design (Korber et al., 2006). The *in silico* drug designing, often referred to as Computer-Aided Drug Design is mainly grouped into two classes which are termed as structure-based and ligand-based. Thus, various techniques are practiced for this to computationally propose a drug or peptide for a particular biological condition. These techniques could either be for designing and discovering purposes or validation. For instance, to discover and recommend a drug for a unique or resistant viral infection, next to the background history and understanding the mechanism of action, a virtual screening (VS) approach is applied that scans various small compounds’ databases (i.e. ZINC, PubChem, MAYBRIDGE, etc.) (Shemetulskis et al., 1995; Irwin and Shoichet, 2005; Kim et al., 2015) to discover those hits that are more likely to be engaged with the target. In the field of Computer-Aided Drug Design, the word “hits” is a term used for compounds that are hypothesized that they may have a stronger affinity with the target. Before this VS technique, a 3D ensemble of chemical and molecular features known as pharmacophore is designed (Schneider et al., 1999; Wadood et al., 2017). It is crucial because this 3D model can recognize similar ligands or macromolecules from the enormous number of drugs like compounds provided in huge databases during the VS process (Sun, 2008).

Apart from VS, molecular docking (MD) (Huang et al., 2006) is a highly acceptable and practiced technique that considers the 3D conformation of a drug and its target in real-time and scores the performance of the drug along with providing various physical and chemical properties. Additionally, it explores how a drug interacts with the target that can be visualized in a 2D or 3D plane. There exists various offline and online software that are used for VS and MD. Few of the major software and servers are GOLD (Joy et al., 2006), AutoDock (Trott and Olson, 2010), PatchDocK (Mehmood et al., 2019; Schneidman-Duhovny et al., 2005; Khan et al., 2018), Molecular Operating Environment (MOE) (Roy and Luck, 2007), Schrödinger suite (Bhachoo and Beuming, 2017), and FireDock (Andrusier et al., 2007). Drugs that perform satisfactorily are observed to be engaged with their targets are subjected to another technique that is termed as molecular dynamics simulation (MDS) which is also a real-time cellular system with an optimum human body pressure, temperature, water, and pH that is built inside a computer driven by a particular forcefield (FF) such as OPLS, AMBER96, GROMACS 431, etc. in software like AMBER (Junaid et al., 2019), GROMACS (Wang et al., 2019) or SCHRÖDINGER (Winstead and Ravaioli, 2003). Nevertheless, the successful outcomes of all these sophisticated techniques (MD and MDS) depend on the most crucial step in this whole process which is the selection of accurate compounds. This selection directly depends on the classical VS technique whose quality is dependent on the quality of the pharmacophore model. Similar to this, QSAR models also bears great importance. It is an approach of vital importance for chemistry and pharmacy that is created on the concept that the activity of a molecule can be altered by bringing amendments into its structural configuration. These structural amendments may be implemented for virtual computational operations, intentional *in vitro* projects such as synthetic research, or only meant to investigate available substances. This method involves the mapping of property and chemical spaces using modeling functions that are associated with a chemical structure to property or more simply a function that relates property to molecular descriptors. This allows us to competently design and propose new potent compounds that possess the required features and perform the desired function.

Researchers in drug designing areas frequently go for existing commercial software like the Schrodinger software suite. This package has in-built modules which are highly demanding like Phase (Dixon et al., 2006a; Dixon et al., 2006b), ConfGen (Watts et al., 2010), and MacroModel (Watts et al., 2010) for Pharmacophore hypotheses generation (Mitra et al., 2010) and 3D QSAR model development (Lee and Briggs, 2001; Kubinyi et al., 2006; Pissurlenkar et al., 2007; Jiang, 2010; Lauria et al., 2010; Puzyn et al., 2010; John et al., 2011). The G protein-coupled receptors (GPCRs) regulate a countless number of physiological signaling cascades in the body, constituting a rich source of targets for the pharmacological deterrence of various human ailments (Atanes et al., 2018). Most of the GPCRs are expressed in the pancreatic islets are still considered as “orphan” which are poorly considered functional and or still have no recommended ligands and have not been used as promising antidiabetic targets (Amisten et al., 2017). A very limited quantity of functionally characterized GPCRs in the human body is the target for greater than 30% of all the current diseases. This stresses the reputation of the rest of GPCRs that lacks a functional chart yet in this regard. Analyses of such targets could reveal innovative ways to treat several biological conditions such as metabolic syndrome, cancer, and diabetes. Concerning the current study, the GPR139 and GPR142 are vital targets for numerous conditions and are therefore targeted in this work to discover potent compounds for them using valid pharmacophore and 3D QSAR studies that may have the ability to prevent us from the situation that is likely caused by these GPCRS.

## Methodology

**FIGURE 1:**
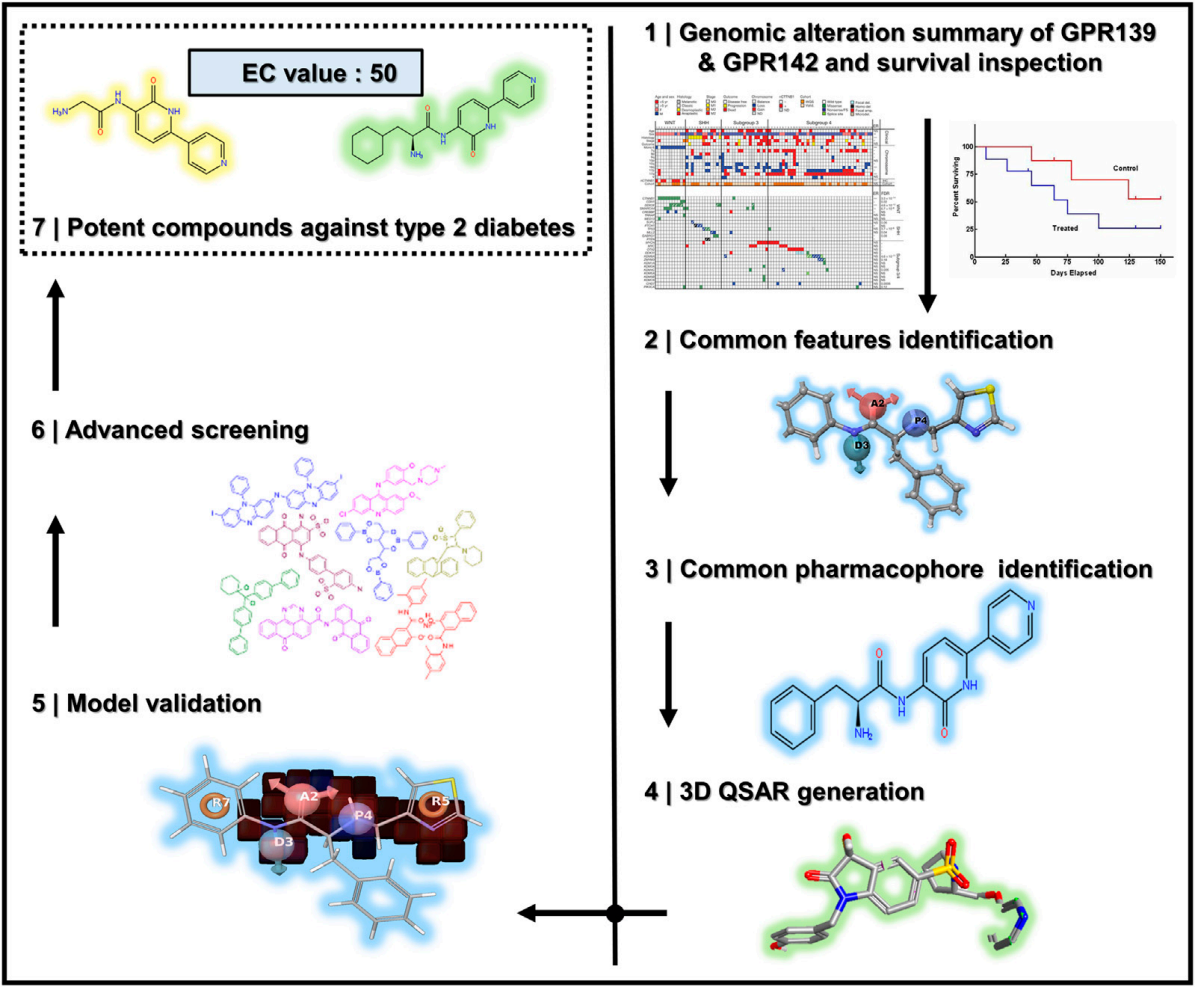
The overall workflow of the steps taken for the successful pharmacophore’s perception, 3D QSAR model development, and 3D database screening for GPR142.

All the steps and techniques employed here are described in detail while the overall workflow of this work is diagrammatically represented in Figure 1.

### Genomic Modifications Summary

The GPR139 and GP142 tumor samples were used to summarize the genomic alterations. For such analysis, wide-ranging CNA (amplifications and homozygous deletions) and color tagging were considered to review alterations in the gene expression. This was an initial approach to comprehend various forms of gene signaling in the Pan-cancer. The common exclusivity and co-occurrence between GPR139 and GPR142 were examined as well. Events associated with a particular cancer are high time differing in tumor clusters i.e., only a solitary biological incident is anticipated to happen in each cancerous sample. An additional situation is the concurrent existence of changes in several genes in the same sample. This was a preliminary way to collect information linked with various gene signaling in the pan-cancer.

### GPR139 and GPR142 Mutations in Pan-Cancer

**FIGURE 2:**
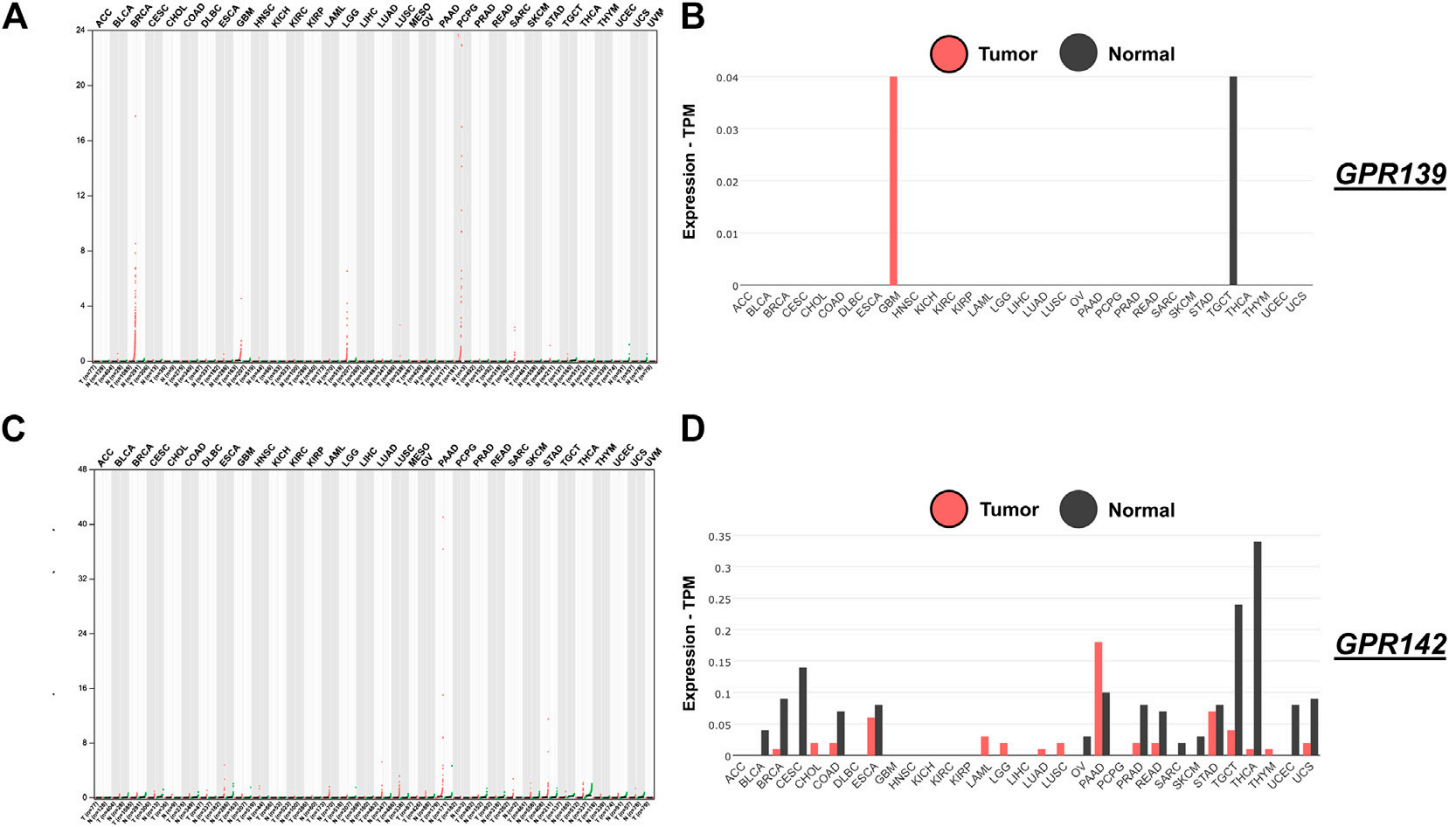
**Expression of** GPR139 and GPR142 in pan-cancer from analyzed TCGA data. **Panel** **(A)** GPR139 expression breakdown; **Panel (B)** GPR139 tumor and normal expression breakdown; **Panel (C)** GPR142 expression study; **Panel (D)** GPR142 tumor and normal expression breakdown.

The locations and frequency of all the mutations within Pfam protein domains were detailed via mutations of GPR139 and GPR142. The whole extent of GPR139 and GPR142 is represented by colored bars while the base of every bar stands for the amino acids’ amount. Colored regions are representing the protein’s domain and the lines and points signify the location and amount of GPR139 and GPR142. The frameshift or nonsense mutations, missense mutations, and in-frames are visually represented in Figure 2.

### Overall Survival Inspection

To highlight changes with time for prognosis purposes, the survival analysis bears great importance. In the current work, the Kaplan-Meier plots were used to evaluate differences in the overall survival among samples that were having more or equal to one alteration as that of the query gene(s). This was applied to those samples also that exhibits no alteration.

### Ligands’ Preparation

First of all, ligands were prepared before the construction of pharmacophore along with the development of a 3D Database using LigPrep (Chen and Foloppe, 2010) which 3D protonates, convert the 2D structure into 3D, form stereoisomers, neutralize charged structures, balances the ionization state and pH using the OPLS2005 force field (Jorgensen and Tirado-Rives, 1988; Jorgensen et al., 1996; Shivakumar et al., 2010).

### Common Pharmacophore Hypotheses Development

A total of 63 compounds (see Supplementary Table S2) from the available literature (Du et al., 2012; Lizarzaburu et al., 2012) were chosen, having a particular EC50 value ranging from 0.036 to 33.00. Ligands were prepared; Amide activity property was selected with all primary properties’ subset. The performance is given as Activity = −log[1*value]. The ligands and their various conformers were classified into sets to be used as input data (three datasets are used in this case). The ligands’ conformer were generated having activity above 0.02 when Active, and activity below 0.01 when Inactive. Conformers were generated in such a way that the number of conformers per flexible bond was equal to 100. The maximum number of conformers per structure was equal to 1,000.

We employed ConfGen for the MacroModel search method that practices rapid sampling. Different conformers were used for Amide bonds which were pre-processed with a minimization step equal to 100 while the minimization step for high energy and redundant conformers was 50. The OPLS2005 force field was employed for this purpose. A maximum relative energy difference equal to 10.0 kcal/mol was maintained for the distance-dependent dielectric that eliminated redundant conformers using the RMSD of 1.0 Å. Structure cleaning- Stereoisomers retain specified chirality, meaning different chiral centers. Thus, the maximum number of stereoisomers kept was 32, and Ionization states retained the original structure using phase Schrodinger suite software (Dixon et al., 2006a).

### Creating Sites

For a given set of pharmacophoric features, sites of each feature in the given ligand conformations were identified and marked such as Acceptor (A), Donor (D), Hydrophobic (H), Negative (N), and Aromatic Rings (R). Features in the vector geometry were edited and a point was selected, in projected acceptor was selected, in point sp3, sp2, and sp were selected, 1 atom was used with distance.

### First Dataset (High-Affinity EC50 Value)

The first dataset contained a total of sixty compounds. The activity thresholds were Active and Inactive if the compounds are above 0.036 and below 0.001 respectively. The maximum activity in the table was 0.930 while the minimum activity is observed as 0.036. The higher and lower number of sites were 7 and 3 and respectively that must resemble a minimum of 35 compounds out of 38 inactive or active’s group. The hypotheses generation scoring, clustering and examined hypotheses, defining excluded volumes, selection of hypotheses for certain QSAR methods as well as search for the similarity or matches with the screened ligands were all carried out.

### Second Dataset (Medium Affinity EC50 Value)

The second Dataset also contained the same number of compounds just like the first one, equal to 60. The activity threshold was Active and Inactive if the compounds are above 1.060 and below 1.000. On the other hand, the supreme movement was 6.6000 while a minimum activity of 1.060. The variants’ list was defined with a total of 7 maximum sites and a minimum of 4 that must match at least 30 compounds out of 51 actives or in active’s group. Score hypotheses generation scoring, clustering and examined hypotheses, defined excluded volumes, selection of hypotheses for certain QSAR methods as well as searching for similar compounds with screened ligands and identified best pharmacophoric featured compounds (Kaushik and Sahi, 2015).

### Third Dataset (Low-Affinity EC50 Value)

The third Dataset was also comprised of 60 compounds, having an activity threshold of >0.036 and <0.035 for Active and Inactive respectively. The maximum activity in the table observed was 33.000 while the minimum activity score was found to be 0.036 that was maintained. Defined variants’ list- the maximum number of sites was 7 and the minimum number of sites was 4 that had to resemble a minimum of twenty compounds with that of the total 60 actives or inactive set. Score hypotheses generation scoring, clustering and examined hypotheses, defining excluded volumes, selection of hypotheses for certain of QSAR methods as well as searching for matching with screened ligands were performed.

## Results

### GPR139 and GPR142 Expression in Pan-Cancer

As a result of the expression analysis of GPR139, it was exposed that a substantial amount of up and down-regulation that explains the hotspots which are responsible for the activation and role in BRCA, GBM, LGG, PCPG, and SARC as shown in Figure 2 while expression analysis of GPR142 revealed a substantial amount of up and down-regulation that explains the hotspots which are responsible for the activation and role in ESCA, LIHC, LUAD, LUSC, PAAD, STAD, TGCT, THCA, and UCS as shown in Figure 2.

### Genomic Site and Variations Summary

**FIGURE 3:**
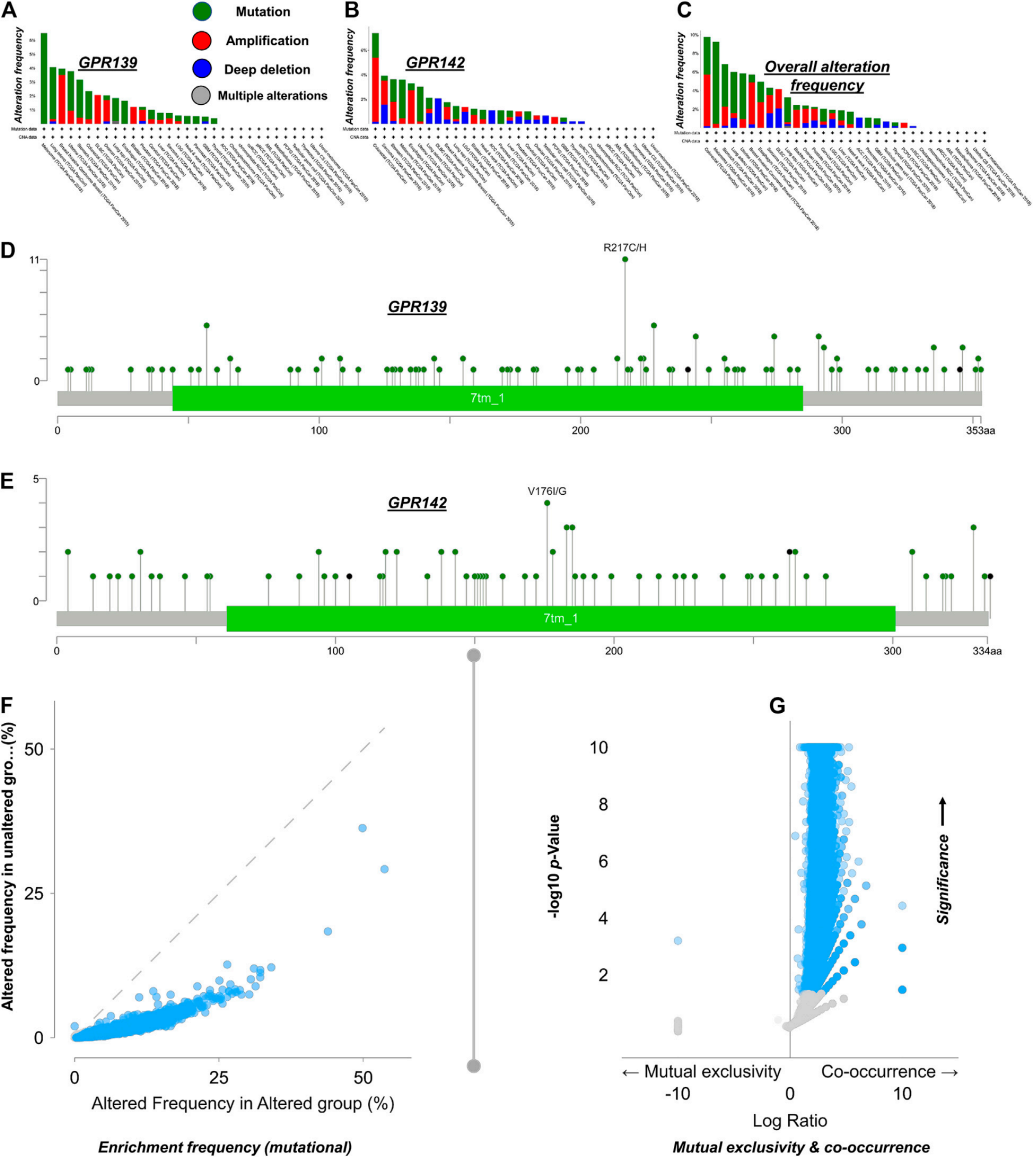
GPR139 and GPR142 Global modification occurrence in pan-cancer based on the TCGA data **Panel (A)** GPR139 variation occurrence investigation; **Panel (B)** GPR142 variation frequency breakdown; **Panel (C)** Inclusive modification occurrence study; **Panel (D)** Ratio of the GPR139 mutations investigation; **Panel (E)**: Amount of GPR142 mutations breakdown. **Panel (F)** Amelioration rate, portraying genomic change per patient in the given samples, showing the mediation of GPR139 and GPR142 signaling in the pan-cancer. Besides, the gene signaling can be facilitated as well upon the instigation or inactivation of cell cycle control through truncating mutations. **Panel (G)** This board illustrates the amount of mutations uniqueness vs co-occurrence in the Genome.

Based on the obtained conclusions, most of the cases were found to be changing the GPR139 and GPR142. Upon further analysis, it was revealed that nearly all of the observed variations were missense mutations. Some deep deletions and few amplifications have also been noticed. Nevertheless, the remaining of the cases experienced alterations in GPR139 and GPR142 that are mainly exhibiting truncating and missense mutations. Examining the mutual exclusiveness suggests that events happened in GPR139 and GPR142 were responsible to occur again in pan-cancer (GPR139 exposed in BRCA, GBM, LGG, PCPG, and SARC) while GPR142 exposed in ESCA, LIHC, LUAD, LUSC, PAAD, STAD, TGCT, THCA, and UCS) as represented in Figure 3.

### Analyzing Survival Rate

**FIGURE 4:**
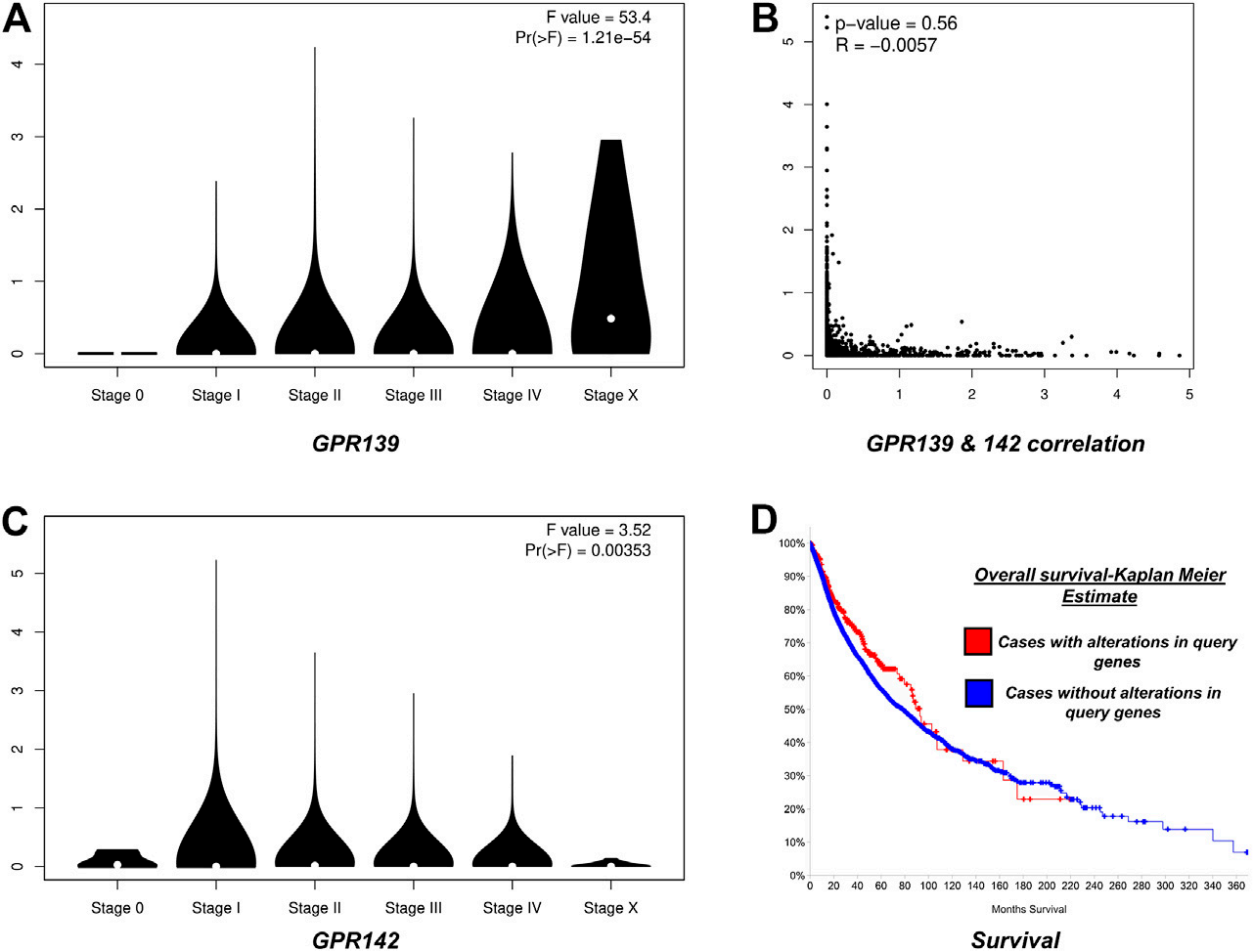
**Panel (A)** Depicts stage plot of GPR139**; Panel (B)**: depicts the correlation between GPR139 and GPR142 in pan-cancer; **Panel (C)**: depicts stage plot of GPR142 and **Panel (D)**: depicts survival analysis of GPR139 and GPR142 in pan-cancer.

To inspect the rate of survival, the Kaplan-Meier approach was used to plot the complete survival analysis for the pan-cancer. Based on the global survival analysis, it was observed that mutations in the cell cycle control were concurrent and were not associated with the overall decreased survival (*p*-value = 0.0615) as illustrated in Figure 4; while correlation analysis of clinical features observed GPR139 and GPR142 correlated in the progression of pathological stages.

### Common Pharmacophoric Features Identification

**FIGURE 5:**
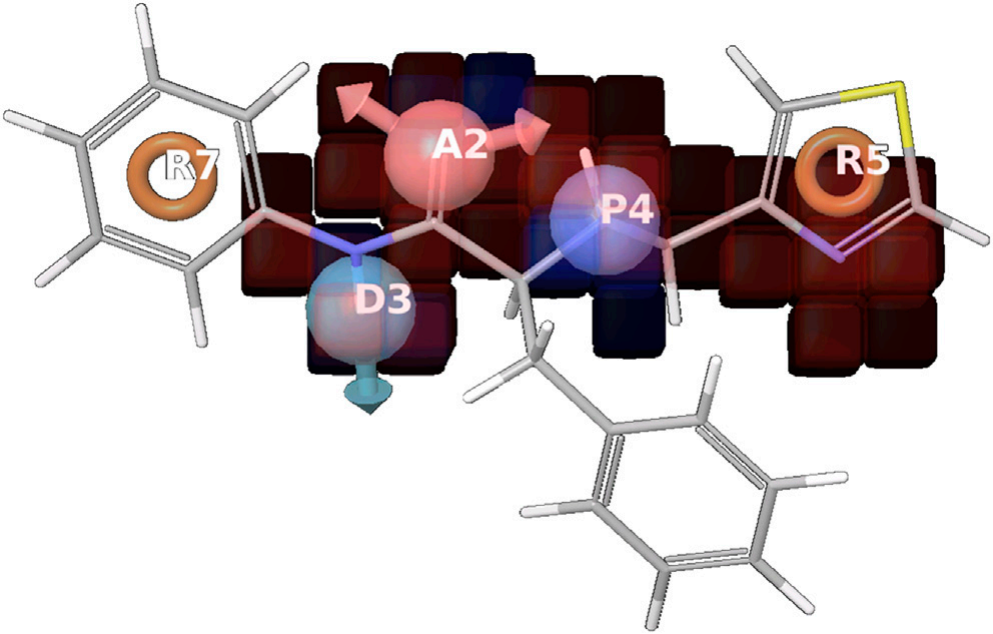
Positive coefficient represented by dark blue color and Negative coefficient represented by the red color, Hydrogen bond donor (D), Hydrophobic/nonpolar (H), Electron-withdrawing (W) shown in the red cube, where R5 has a common pharmacophoric feature, responsible for the activity. A2 are in the negative coefficient and other R7, D3P4, and R5 are in the positive coefficient shown in the supplementary information (Supplementary Figure S2).

Maintaining common features in a pharmacophore model is very important as they are the backbone of the parent molecules on which the functionality of the drug highly depends. All the common pharmacophoric features obtained from the 3D Database of GPR142 using phase Schrodinger suite software are summarized in the Supplementary Table S1. Out of 1,038 chemical structures from GPR142 3D Database; only 5 are chosen that are flexible chemical structures with an average reduction of up to 1.18 Å plus they are having a better common pharmacophoric match for pan-cancer target pharmacophore shown in Figure 5.

### Common Pharmacophore Identification

To identify a common pharmacophore model, the selected and desired variants were used for the given active ligands (Tables 1,2).

**TABLE 1:** Pharmacophoric variant listing 21 out of 21 were selected, where the highest and lowest amount of positions are 7 and 4 correspondingly.

AAAADDP	AAAADDR	AAAADPR	AADPRRR	AAAADRR	DDPRRRR	AAPRRRR
AAAAPRR	AAAARRR	AAADDPR	AADDRRR	AAADDRR	ADPRRRR	ADDPRRR
AAADPRR	AAADRRR	AAAPRRR	AADDPRR	AAARRRR	ADDRRRR	AADRRRR

The selected variants were used for the generation of a maximum number of pharmacophoric hypotheses derived from a common pharmacophore model for the variants among the given active ligands. All the 7 out of 7 variants accompanying the number of maximum pharmacophoric features were selected.

**TABLE 2:** Selected and desired variants finding common pharmacophore for the variants among the given active ligands, 7 out of 7 selected variants can be seen in the variants’ column.

Variant	Maximum number of hypotheses
ADRRR	1
AADPR	5
AAPRR	8
APRRR	7
DPRRR	6
ADPRR	22
AADRR	3

### Score Hypotheses

Score hypotheses generation was created by 30 out of 51. Apart from this, clustering and examination of the hypotheses, defining excluded volumes, and the selection of hypotheses for certain QSAR methods as well as the search for features with screened ligands were carried out. Score Actives; vector and site filtering maintained only those variants’ that are having RMSD below 1.200 Å, vector score above 0.500 Å, and be the top 10%. The quantity should be in the range of 10 (minimum) and 50 (maximum). The survival score formula for vector score is 1.000, +1.000 for site score, +1.000 as volume score, −0.000 for the reference ligand relative conformational energy, +0.000 as selective score, +1.000 for the number of matches, and +0.000 in case of the reference ligand activity. All the values are listed in Table 3.

**TABLE 3:** Score hypotheses of ADPRR.18 where site, volume, and matches are listed with alignment for a hypothesis of ADPRR.18 where compound number 25 is observed to be a more valid and suitable one.

	Site		Vector	Volume		Selectivity	Match		Survival	Energy		Activity
**ADPRR.18**	0.71		0.913	0.611		2.273	31		3.237	0.000		3.300
**Activity**		**Pharm set**			**Fitness**			**Site matched**			**Relative energy**		
3.300			Active		3.00			5			0.000		

### Building QSAR Model

In QSAR (Quantitative structure-activity relationship) model building, the predicted structure-activity relations for the matching ligands are investigated. The training set where the random seed was 0, keeping actives and inactive in the training set. The 3D QSAR model parameters were kept as standard. For example, the Grid sampling was 1.00 Å, the maximum PLS factor was 1, and the model type was atom-based (Table 4).

**TABLE 4:** Training set where the random seed was 0, keeping actives and inactive in the training set where grid sampling is equal to 1.00 Å, maximum PLS factor was 1 and the model type is atom-based.

QSAR set (Training)	QSAR set (Test)
66, 64, 63, 62, 61, 60, 59, 58, 57, 56, 55, 53	54, 14, 13, 7, 6, 5, 4, 3, 2
51, 48, 45, 43, 42, 40, 39, 35, 34, 33, 32, 31	
30, 29, 27, 26, 24, 23, 22, 21, 20, 19, 18, 17	
16, 15, 52, 50, 49, 46, 41, 36, 28, 25, 11, 10, 9, 8, 1	

### Model Validation

**FIGURE 6:**
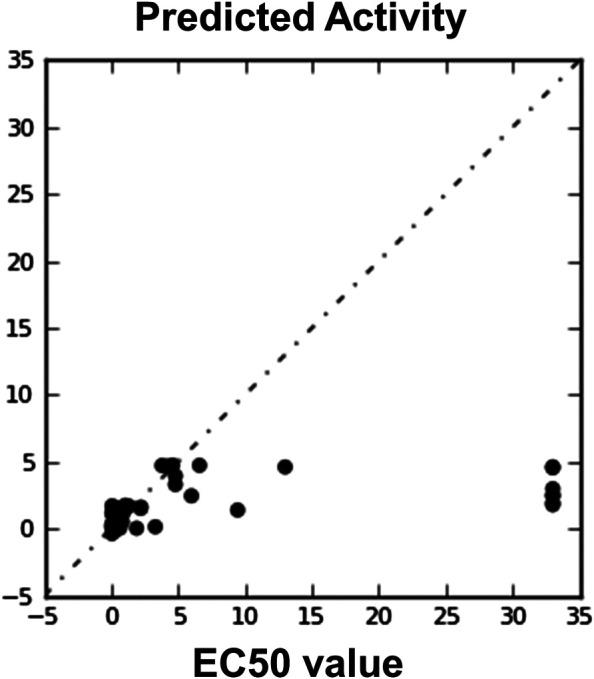
Automatically generated regression plot of 3D QSAR EC50 value and phase predicted activity, where the *X*-axis signifies the EC50 score and *Y*-axis denotes the phase predicted activity of the chemical structure.

The model validation phase of the 3D QSAR models uses distinct training and testing sets for the cross-validation approach. The 3D QSAR models estimate outcome which is derived from the training set shown in Figure 5. The highly stable models are preferred as they don’t rely completely on the training set. Regarding the statistical parameters for the training set, the m represents the number of PLS factors in the model, n represents the number of molecules in the training set, dʄ1 = m + 1 represents the degrees of freedom in the model, dʄ2 = n – m – 2 denotes the degree of freedom in the data, y represents the detected movement for a training set molecule i, yˆ_i_ signifies the predicted activity for training set molecule i and *R* ^2^ = 1–σ^2^/σ^2^ is the R-squared or coefficient of determination. Test set prediction was performed by phase defined parameters, where T signifies the test set of molecules, n_T_ denotes the number of molecules in T, Y_j_ is the observed activity for molecule j ε T, yˆ_j_ means the predicted activity for molecule j ε T, and Q^2^ = *R* ^2^ (T) represents the Q-squared. The necessary parameters for the ADPRR are given in Table 5; Figures 5,6.

**TABLE 5:** 3D QSAR model parameters for ADPRR.18 pharmacophoric hypotheses, where R-squared value is 0.6478 which is considered a more favorable pharmacophoric feature in this case.

ID	SD	R-squared	F	P	Stability	RMSE	Q-squared	Pearson-R
ADPRR.18	1.0282	0.6478	90.1	1.097e-12	0.9449	26.7754	-7.674	0.0504

### Screened Ligands and Known Compounds’ Pharmacophore Analysis

**FIGURE 7:**
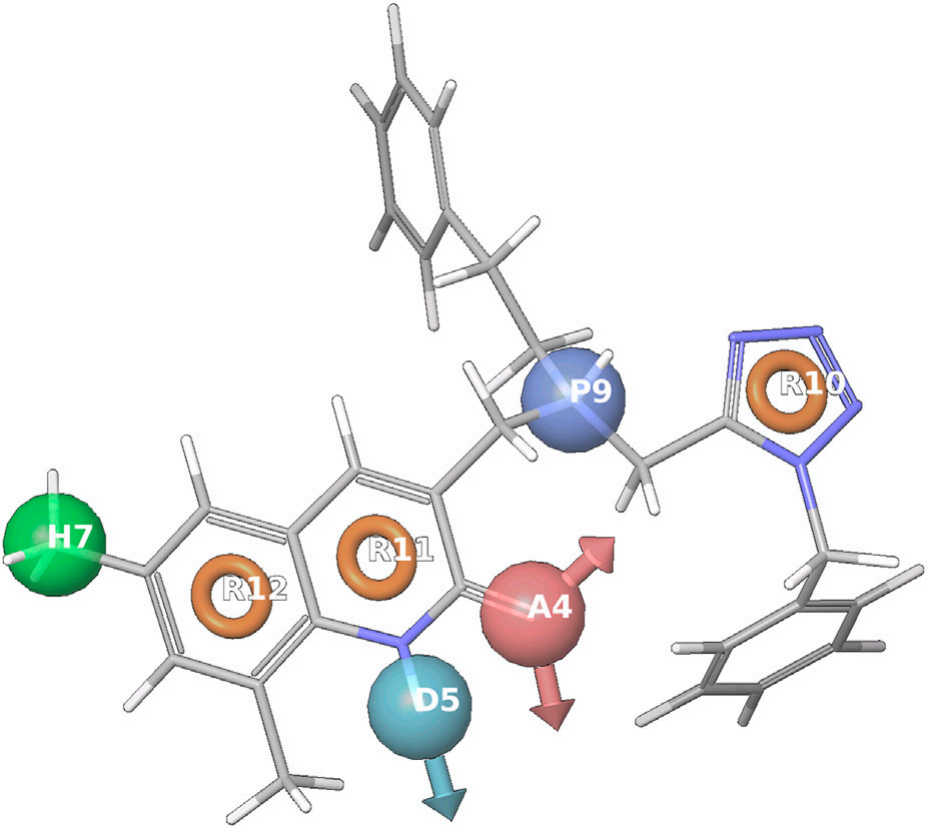
Represents the common pharmacophore hypotheses using screened ligand and Known Compounds, where R10 has the most important common pharmacophoric feature that inhibits cancer.

**FIGURE 8:**
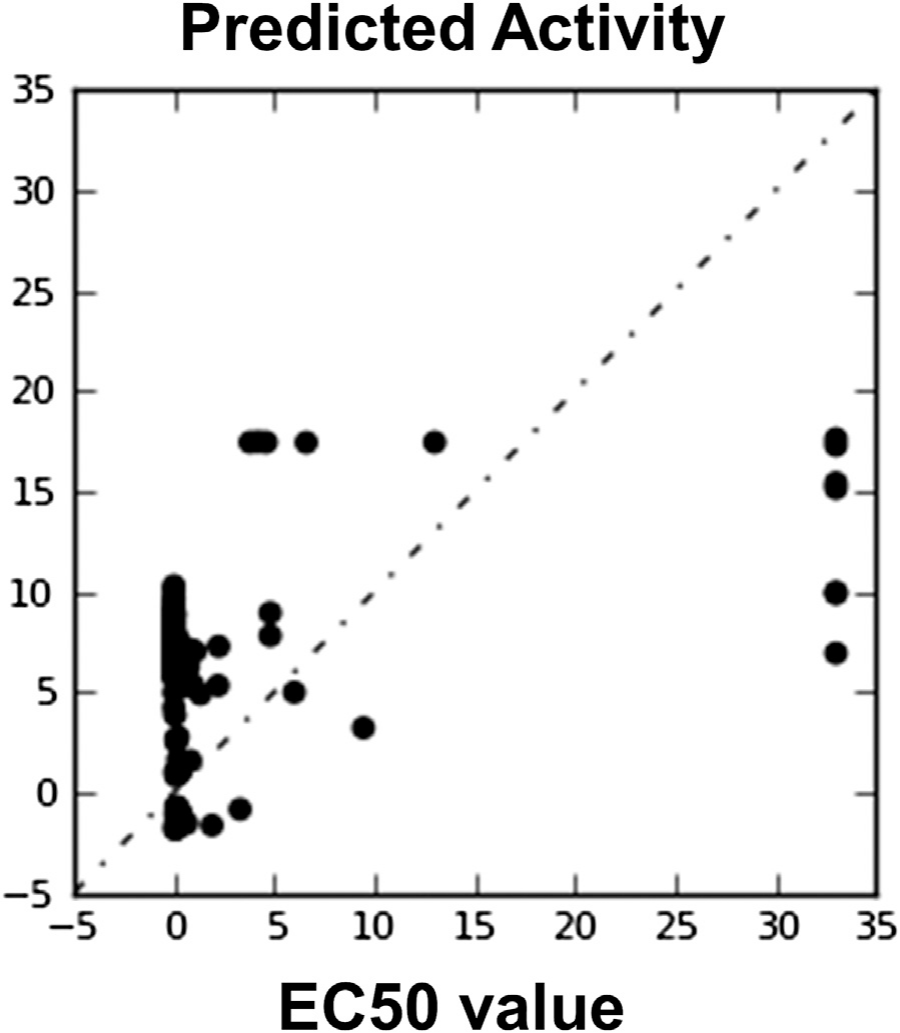
Automatically generated regression plot of 3D QSAR known EC50 value and phase predicted activity, where *X*-axis signifies the EC50 score activity and *Y*-axis signifies the phase predicted activity of chemical structure.

The common pharmacophore using screened and known EC50 compounds was found along with a pharmacophore for those variants which are among the given active ligands. The variants’ list was defined while the higher and the lower quantity regarding the sites were kept as 7 and 4 correspondingly, matching at least 20 compounds out of 137 active or inactive groups as shown in Figure 7. The variant list 269, after the identification of common pharmacophore, the ADHPRRR.35 was considered having maximum hypotheses as 16 with a survival rate of 2.960. The site score was 0.58, a vector was equal to 0.939, volume was 0.442, selectivity was 3.858 and the number of matches was 22. After the alignment of ADHPRRR.35 hypotheses, its fitness value turned out to be 2.02 and the number of site matches was 7 listed in Table 6; Figures 7,8.

**TABLE 6:** 3D QSAR model parameters for ADHPRRR.35 pharmacophoric hypotheses where grid sampling was 1.00 Å, maximum PLS factor was 1, and the model type was an atom-based pharmacophoric analysis.

ID	SD	R-squared	F	P	Stability	RMSE	Q-squared	Pearson-R
ADHPRRR.35	8.5856	0.3407	30	9.84e-07	0.931	7.6838	0	0

### New Pharmacophore Hypotheses Generation Using Glide Docking Score

The compounds were docked and scored by the newly identified common pharmacophore to find common features for the variants present among the given active ligands. Defining the variants’ list and the highest and lowest number of spots as 7 and 4 correspondingly that must resemble a minimum of 95 compounds out of 137 active/inactive groups was done and maintained. After the identification of a common pharmacophore model, the variants’ list was 24 where R8 is common in all pharmacophoric analysis and is observed to be responsible for the native activity of compounds, given in the supplementary information (Supplementary Figure S1).

### Building QSAR Model

**FIGURE 9:**
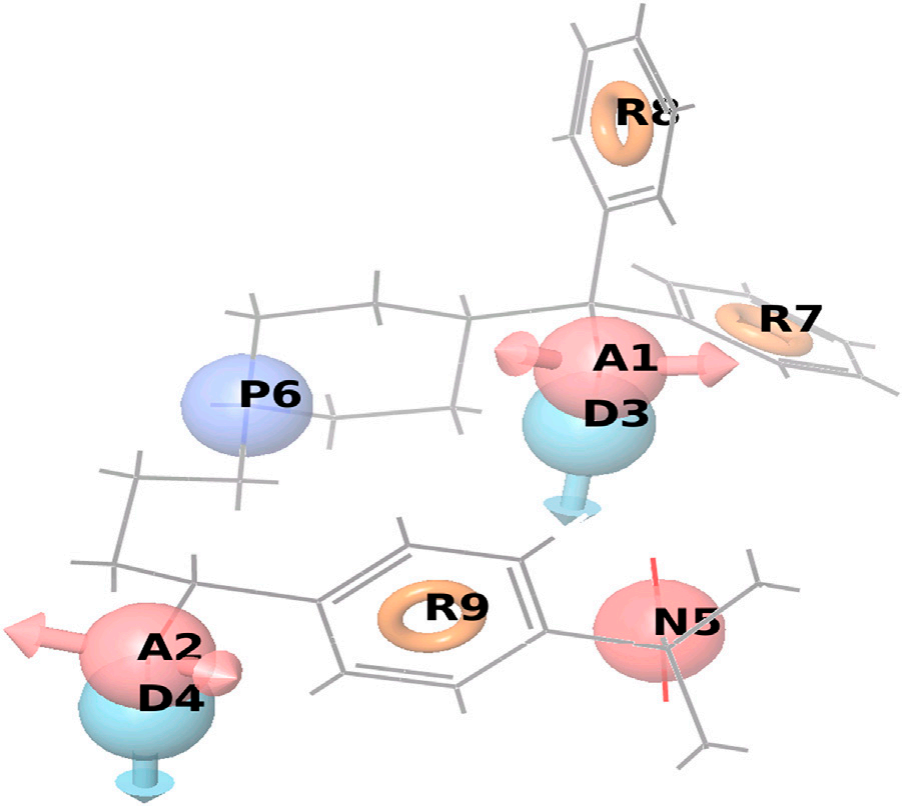
Represents the common pharmacophore hypotheses using the docking score of screened ligands, where R8 has the most important common pharmacophoric feature that inhibits cancer.

The QSAR model was build and examined for the predicted structure-activity relations concerning the matching ligands. The random seed in the training set was 0, keeping both the actives and inactive in the training set, sampled uniformly over the activity coordinates. The random training set was only 50%, 3D QSAR model parameters Grid sampling was 1.00 Å, maximum PLS factor was 1, and the model type was atom-based. After the alignment, Compound6 alignment score was observed to be 0.089967, vector score was equal to 0.996997, volume score was given as 0.428221, fitness value of 2.350246 and phase predicted score was -4.86423, Compound7 alignment score was 0.095122, vector score as 0.936649, volume score was observed to be 0.37549, fitness value was given as 2.232871 and phase predicted score was equal to −5.10154. Similarly, the Compound8 alignment score was 0.298618, vector score was 0.989711, volume score turned out to be 0.427929, fitness value as 2.168791 and phase predicted score equal to −5.16605 as shown in Figure 9.

### Advanced Pharmacophore Screening of GPR142

Various hypotheses assisted in the identification of new matches and the conformers were generated automatically. For the conformer’s generation and refinement, existing conformers were discarded, the number of structures per adjustable bond was kept as ten, the highest number of conformers per assembly was 100 with rapid sampling. The relative energy window was equal to 10.0 kcal/mol. Conformer’s generation was skipped in case of rotatable bonds greater than fifteen. The matching tolerance was 2.0 Å in the inter-site and must match on at least 4 site positions out of 4. For the hit treatment purposes, hits were sorted out by decreasing fitness, that returned 1,000 hits in total, i.e. 1 hit per molecule. Fitness of hit treatment was equal to 1.0 (for alignment score/1.2) + 1.0 (for vector score) and + 1.0 volume score. All hits with < −1.0 vector score and <0.0 volume score were rejected.The **Compound1, Compound2, and Compound3** were obtained after screening from the 3D database of GPR142, having the same pharmacophoric features that inhibit cancer.

### Advanced Pharmacophore Screening of Known EC50 Compounds and Screened Compounds

Advanced pharmacophoric screening of known EC50 compounds and Screened Compounds was done using the default parameters. The existing conformers were discarded, the number of structures per adjustable bond was kept ten. The highest number of conformers apiece structure was kept as 100 with rapid sampling. The relative energy window was 10.0 kcal/mol, skipping conformer generation for structures with >15 rotatable bonds. Matching in inter-site distance matching tolerance was maintained at 2.0 Å and must match on at least 4 site positions out of 4. For the case of hit treatment purpose, hits by decreasing fitness turned out 1,000 in total which is equivalent to 1 hit per molecule. Fitness of the hit treatment was 1.0 (for alignment score/1.2) + 1.0 (for vector score) + 1.0 volume score. All hits with < −1.0 vector score and < 0.0 volume score were totally rejected. **Compound4 and compound5** were obtained after screening the 3D Database of GPR142, which has the same pharmacophoric features and common chemical structures that can inhibit cancer.

### Advanced Pharmacophore Screening and Analysis of New Pharmacophore Model

Advanced pharmacophoric screening of the newly identified pharmacophore model was performed by the glide docking scoring system. The number of conformers was set per rotatable bond as 10, a maximum number of conformers per structure was 100 with rapid sampling. The relative energy window was 10.0 kcal/mol. All the conformers’ generation in case of structures with >15 rotatable bonds were skipped. The matching in inter-site distance matching tolerance was kept as 2.0 Å and must match on four sites position out of 4. Hits were sorted by decreasing fitness returning at its most equal to1000 hits in total that is 1 hit per molecule. The fitness of hit treatment was 1.0 (for alignment score/1.2) + 1.0 (for vector score) + 1.0 volume score. Reject hits with < −1.0 vector score and < 0.0 volume score. In all the considered compounds, **Compound6, compound7, and Compound8** were chosen after screening the 3D Database of GPR142. They all were having the same pharmacophoric features and can inhibit cancer.

### Advanced Pharmacophore Screening of GPR139

The Paralog of the GPR142 gene is GPR139, and the similarity gene and protein between GPR139 and GPR142 is 50%, sharing suggested ligands. The advanced pharmacophoric screening was carried out by these protocols such as; finding matches from the generated features' search during conformers exploration that were automatically generated. All existing conformers were discarded, the conformers apiece adjustable bond was chosen to be 10. On the other hand, the maximum amount for conformers apiece structure was chosen to be 100, the relative energy window was equal to 10.0 kcal/mol. Similarly, conformers’ generation for structures with greater than 15 rotatable bonds was skipped. Matching in inter-site distance matching tolerance was 2.0 Å and must match on at four site positions out of the total 4. All hits were sorted decreasing the fitness value, returning at the most given hits which are 1,000 in total that is 1 hit per molecule. Fitness of hit treatment was 1.0 (for alignment score/1.2) + 1.0 (for vector score) + 1.0 as the volume score. Hits were rejected with < −1.0 vector score and < 0.0 volume score. The **Compound9 and Compound10** were selected as a result of screening from the 3D Database of GPR139, which contains similar pharmacophoric features like GPR142 target compounds. Compound9 and Compound10 might be helpful in the proteins’ dimerization activity and neuropeptide receptor activity as provided in the supplementary data (Supplementary Table S3).

## Conclusion

Computational drug design mainly emphasizes structure-based techniques. The *in silico* screening of small compounds in large databases using developed pharmacophoric features is a good way to discover and identify the best chemical compounds by using common pharmacophoric hypotheses generation and 3D QSAR models. However, extra care should be taken during the development of such a model as the performance of the predicted compounds depends on the features considered. It is a more feasible, robust, and flexible way to use such models. The current study reports on genomic alterations of GPR139 and GPR142 through tumor samples, assisting in the identification of common drug compounds for GPR142 target by 3D database by scanning common pharmacophoric features and experimental EC50 value which might be useful in the inhibition of cancer. We intend to subject these compounds to explore their interactions with the target and stability in the host region via long term molecular dynamics simulation and *in vitro* analysis.

## Data Availability

All the datasets analyzed during this study are available from the corresponding authors upon a reasonable request.
