# Supplementary material for: Pan-Cancer Analysis and Drug Formulation for GPR139 and GPR142
Source: Front Pharmacol. 2021 Feb 19;11:521245. doi: 10.3389/fphar.2020.521245 (PMC7933564; doi:10.3389/fphar.2020.521245)
Supplement: Supplementary file 1 [file datasheet1.docx]

**Pan-cancer Analysis and Drug Formulation for GPR139 and GPR142**

**Aman Chandra Kaushik^1#^ | Aamir Mehmood^2,3#^ | Xiaofeng Dai^1^ | Dong-Qing Wei^2,3 *^**

^1^Wuxi School of Medicine, Jiangnan University, Wuxi, China

^2^School of life Sciences and Biotechnology, Shanghai Jiao Tong University, 800 Dongchuan Road, Shanghai 200240

^3^Peng Cheng Laboratory, Vanke Cloud City Phase I Building 8, Xili Street, Nanshan District, Shenzhen, Guangdong, 518055, P.R China

**SUPPLEMENTARY INFORMATION**

**TABLE S1** Top10 compounds of GPR142 after screened from 3D Database of GPR142 as well as GPR139, which have same pharmacophoric features

| 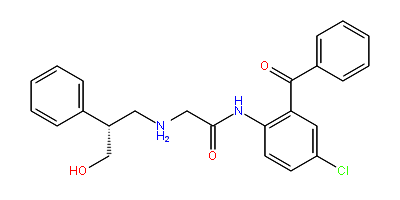  **Compound1** | 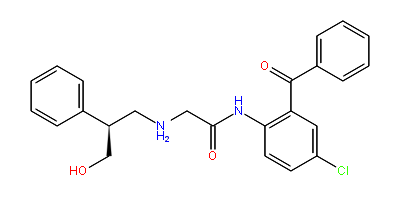  **Compound2** |
| --- | --- |
| 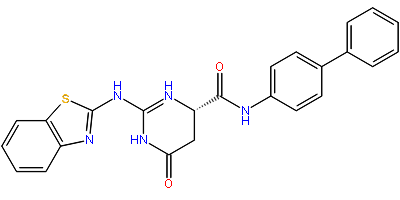  **Compound3** | 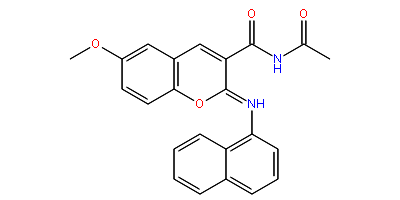  **Compound4** |
| 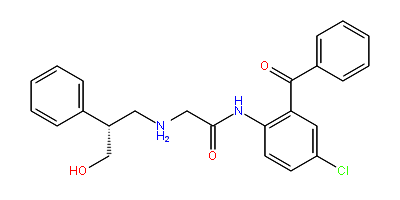  **Compound5** | 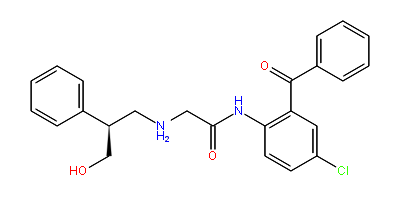  **Compound6** |
| 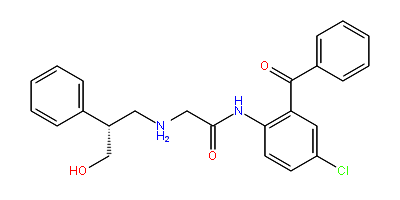  **Compound7** | 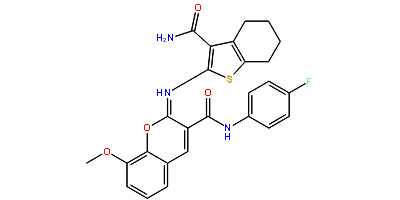  **Compound8** |
| 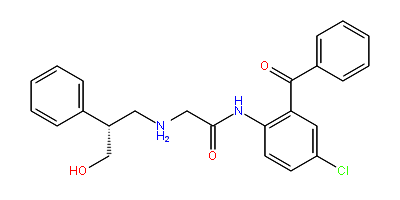  **Compound9** | 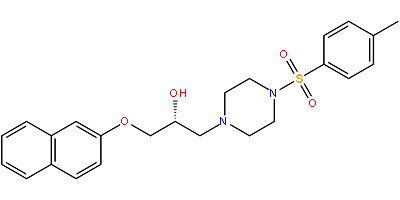  **Compound10** |

**TABLE S2** represents the activity of developed common pharmacophore hypotheses using PHASE Schrodinger suite software, from experimental EC50 value of chemical structure from the literature. Where plus (+) sign are represents the activity of compounds and minus (-) sign represents the inactivity of compounds. And more the one plus signs are indicating more active compounds.

| **Compounds** | **Experimental Activity (EC50)** | **Structure** | **Activity** | **Screened Compounds** | **Predicted Activity** |
| --- | --- | --- | --- | --- | --- |
| 01 | 4.8 | 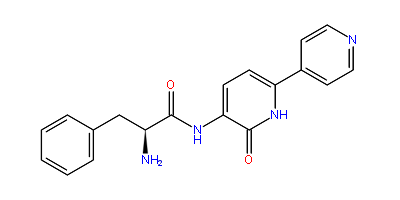 | + | Compound01 | +++ |
| 02 | 13 | 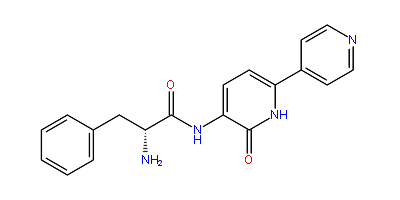 | - | Compound02 | +++ |
| 03 | >33 | 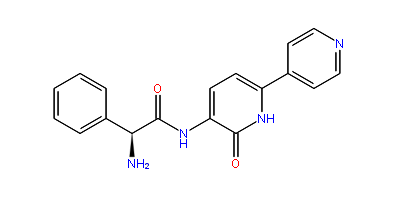 | - | Compound03 | +++ |
| 04 | >33 | 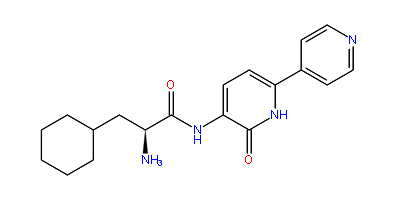 | - | Compound04 | +++ |
| 05 | >33 | 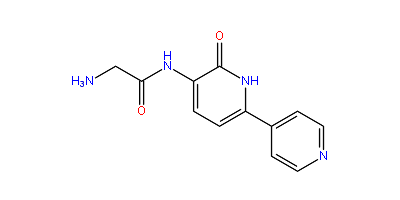 | - | Compound05 | +++ |
| 06 | >33 | 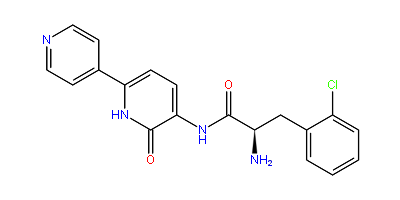 | - | Compound06 | +++ |
| 07 | >33 | 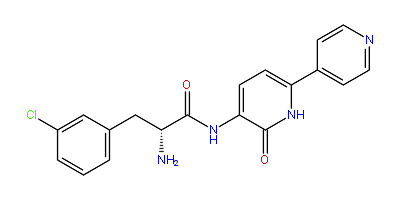 | - | Compound07 | +++ |
| 08 | 3.8 | 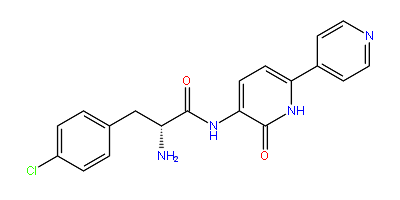 | + | Compound08 | +++ |
| 09 | 4.2 | 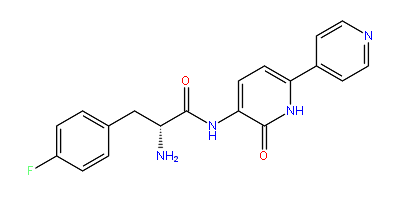 | + | Compound09 | ++ |
| 10 | 4.6 | 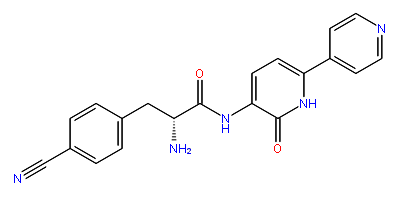 | + | Compound10 | +++ |
| 11 | 6.6 | 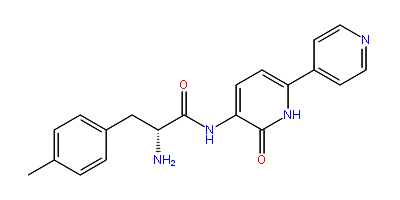 | + |  |  |
| 12 | 25 | 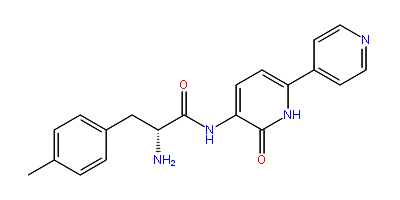 | - |  |  |
| 13 | >33 | 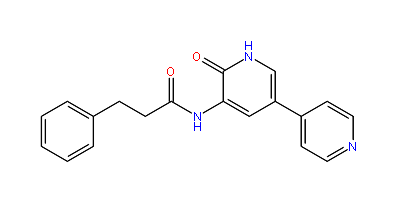 | - |  |  |
| 14 | >33 | 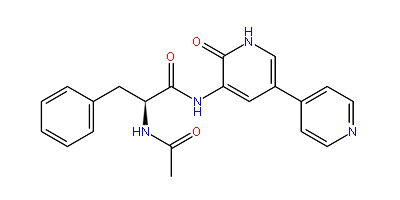 | - |  |  |
| 15 | 0.76 | 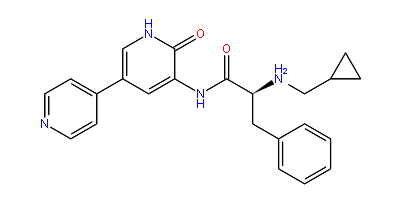 | +++ |  |  |
| 16 | 0.83 | 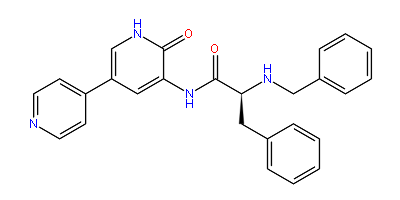 | +++ |  |  |
| 17 | 0.78 | 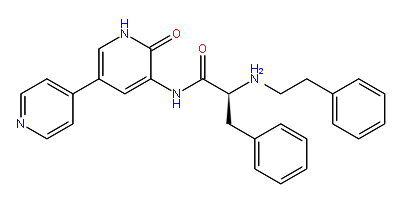 | +++ |  |  |
| 18 | 0.093 | 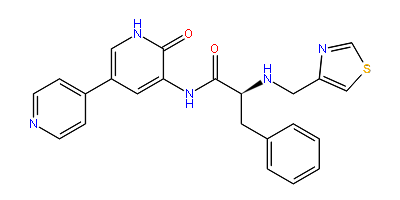 | +++ |  |  |
| 19 | 0.36 | 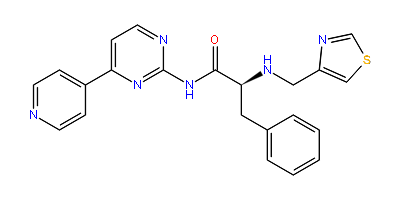 | +++ |  |  |
| 20 | 0.088 | 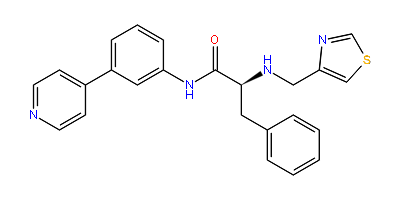 | +++ |  |  |
| 21 | 0.089 | 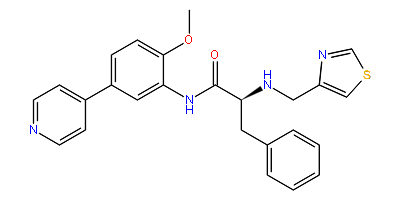 | +++ |  |  |
| 22 | 0.053 | 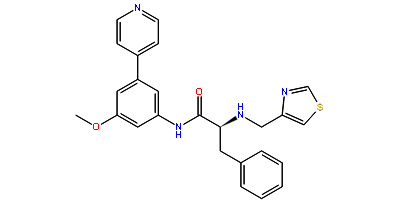 | +++ |  |  |
| 23 | 0.24 | 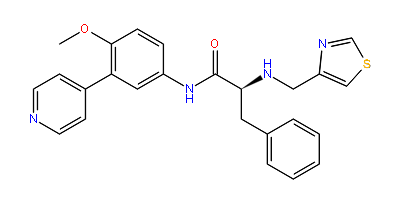 | +++ |  |  |
| 24 | 0.11 | 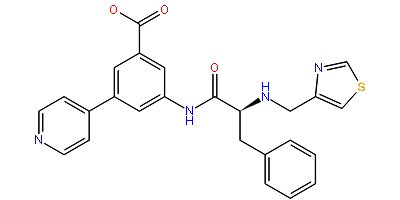 | +++ |  |  |
| 25 | 3.3 | 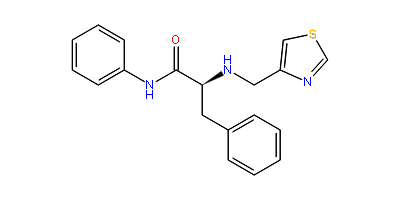 | ++ |  |  |
| 26 | 0.052 | 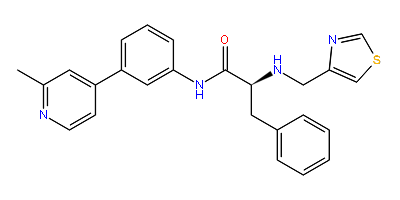 | +++ |  |  |
| 27 | 0.21 | 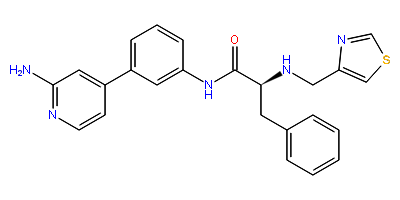 | +++ |  |  |
| 28 | 1.9 | 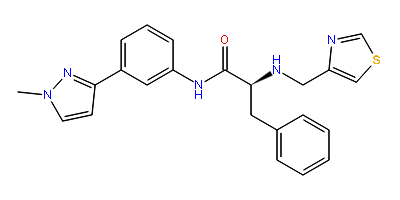 | ++ |  |  |
| 29 | 0.067 | 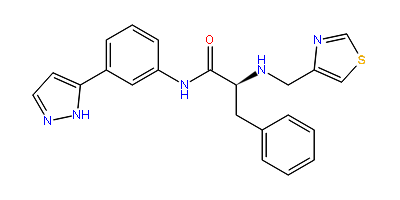 | +++ |  |  |
| 30 | 0.18 | 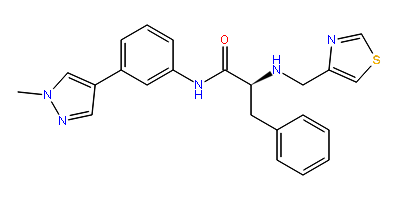 | +++ |  |  |
| 31 | 0.18 | 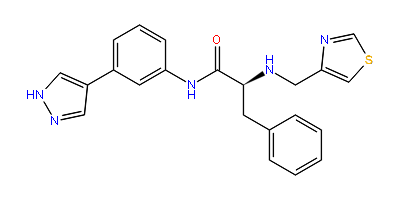 | +++ |  |  |
| 32 | 0.64 | 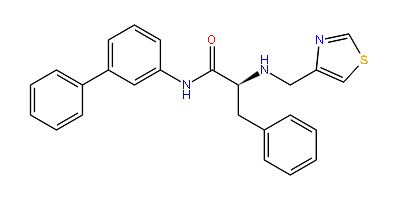 | +++ |  |  |
| 33 | 0.067 | 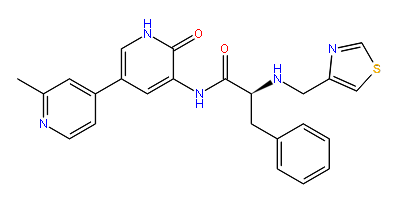 | +++ |  |  |
| 34 | 0.20 | 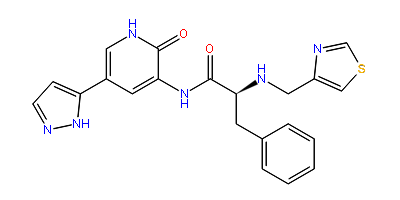 | +++ |  |  |
| 35 | 0.23 | 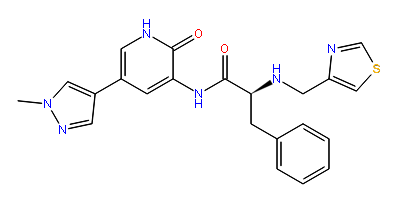 | +++ |  |  |
| 36 | 4.8 | 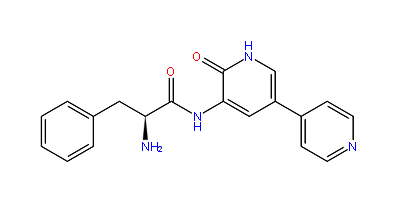 | + |  |  |
| 39 | 0.067 | 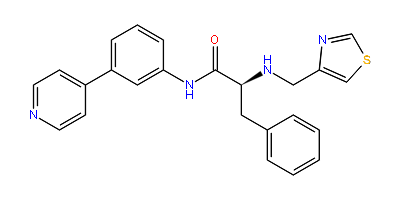 | +++ |  |  |
| 40 | 0.93 | 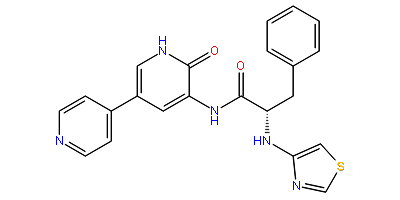 | +++ |  |  |
| 41 | 2.2 | 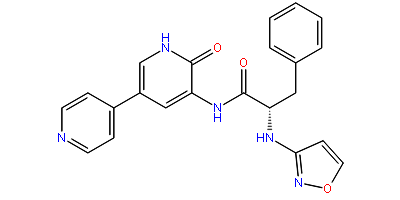 | ++ |  |  |
| 42 | 0.11 | 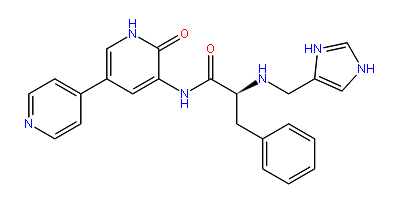 | +++ |  |  |
| 43 | 0.21 | 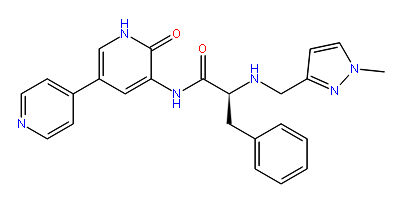 | +++ |  |  |
| 44 | 0.095 | 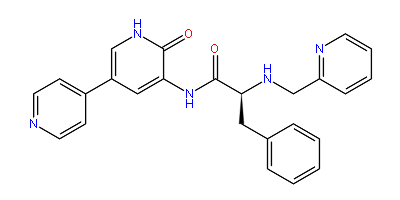 | +++ |  |  |
| 45 | 0.44 | 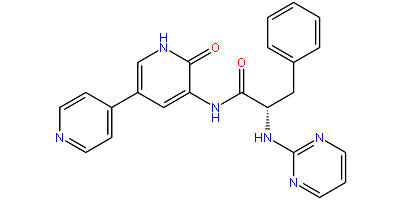 | +++ |  |  |
| 46 | 1.32 | 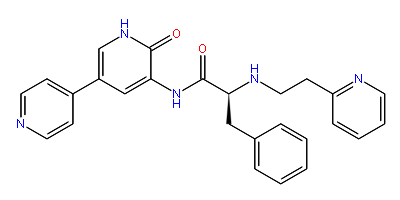 | ++ |  |  |
| 48 | 0.39 | 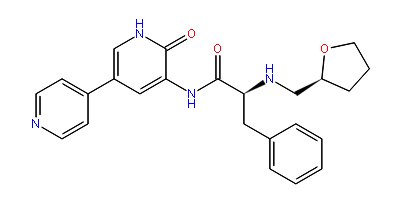 | +++ |  |  |
| 49 | 1.06 | 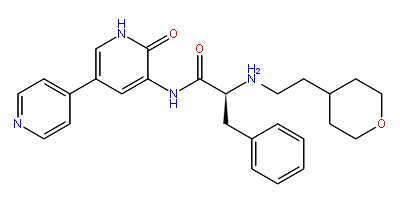 | ++ |  |  |
| 50 | 2.23 | 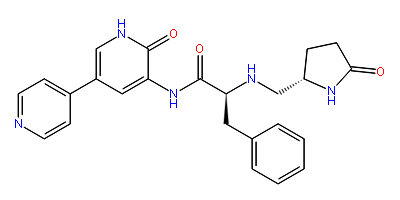 | + |  |  |
| 51 | 0.22 | 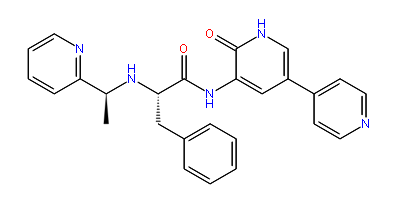 | +++ |  |  |
| 52 | 6.0 | 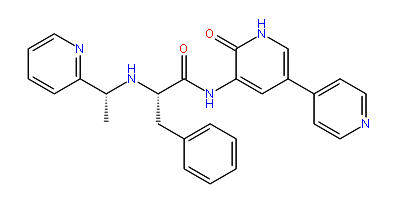 | - |  |  |
| 53 | 0.73 | 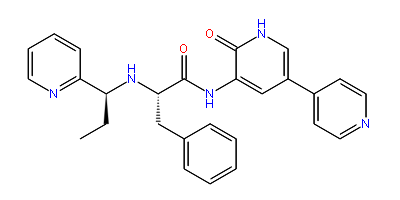 | +++ |  |  |
| 54 | 9.45 | 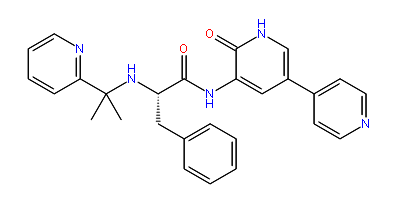 | - |  |  |
| 55 | 0.054 | 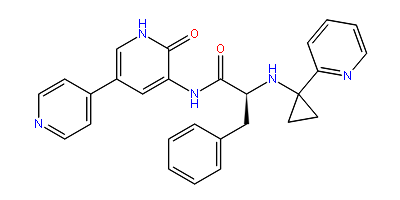 | +++ |  |  |
| 56 | 0.099 | 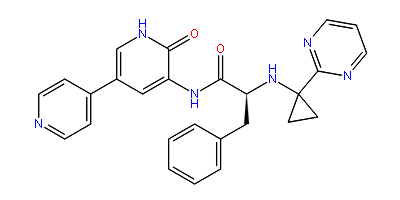 | +++ |  |  |
| 57 | 0.20 | 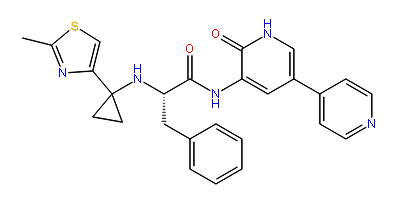 | +++ |  |  |
| 58 | 0.26 | 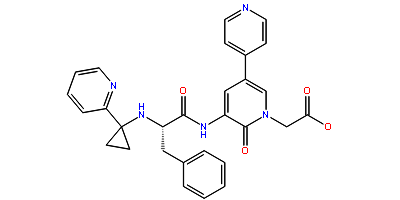 | +++ |  |  |
| 59 | 0.28 | 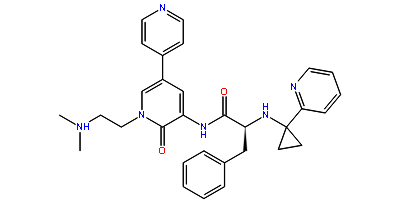 | +++ |  |  |
| 60 | 0.35 | 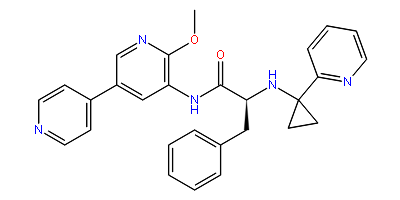 | +++ |  |  |
| 61 | 0.086 | 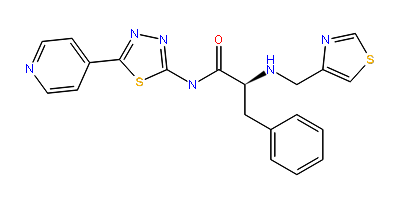 | +++ |  |  |
| 62 | 0.036 | 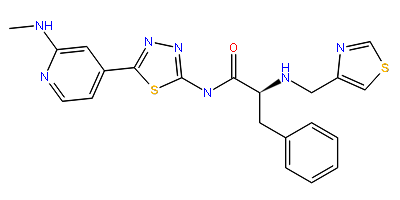 | +++ |  |  |
| 63 | 0.18 | 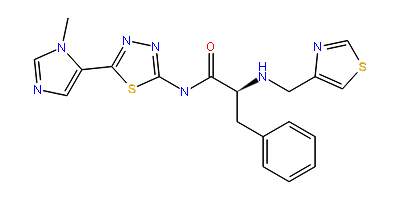 | +++ |  |  |
| 64 | 0.39 | 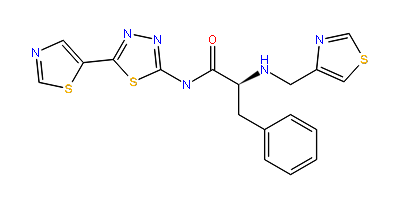 | +++ |  |  |
| 66 | 0.095 | 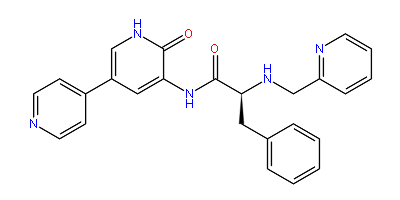 | +++ |  |  |

**TABLE S3** Top10 compounds of GPR142 after screened from 3D Database, which has the same pharmacophoric features, where plus signs represent the activity of compounds.

| **Compounds** | **Molecular Formula** | **Docking Score (VSW)** | **Activity** | **Donor**  **HB** | **Accept**  **HB** | **Molecular Weight** | **Percent of Human Oral Absorption** | **XLogP3** | **QPlog**  **Po/w** |
| --- | --- | --- | --- | --- | --- | --- | --- | --- | --- |
| Compound1 | C_24_H_24_ClN_2_O_3_^+^ | -6.542 | +++ | 2.000 | 6.700 | 423.911g/mol | 92.100 | 4.3 | 4.247 |
| Compound2 | C_24_H_24_ClN_2_O_3_^+^ | -7.176 | +++ | 2.000 | 6.700 | 423.911g/mol | 91.966 | 4.3 | 4.254 |
| Compound3 | C_24_H_18_N_5_O_2_S^-^ | -6.470 | +++ | 3.000 | 8.000 | 440.497g/mol | 93.391 | 4.8 | 3.764 |
| Compound4 | C_23_H_18_N_2_O_4_ | -6.807 | +++ | 1.000 | 5.250 | 386.400g/mol | 100.000 | 4.2 | 4.503 |
| Compound5 | C_24_H_24_ClN_2_O_3_^+^ | -6.542 | +++ | 2.000 | 6.700 | 423.911g/mol | 92.100 | 4.3 | 4.247 |
| Compound6 | C_24_H_24_ClN_2_O_3_^+^ | -7.176 | +++ | 2.000 | 6.700 | 423.911g/mol | 91.966 | 4.3 | 4.254 |
| Compound7 | C_24_H_24_ClN_2_O_3_^+^ | -6.542 | +++ | 2.000 | 6.700 | 423.911g/mol | 92.100 | 4.3 | 4.247 |
| Compound8 | C_26_H_23_FN_3_O_4_S^+^ | -6.177 | +++ | 3.000 | 7.250 | 492.5419g/mol | 100.000 | 5 | 4.198 |
| Compound9 | C_24_H_24_ClN_2_O_3_^+^ | -8.911 | ++ | 2.000 | 6.700 | 423.911g/mol | 74.056 | 4.3 | 4.247 |
| Compound10 | C_24_H_28_N_2_O_4_S | -7.450 | +++ | 1.000 | 8.950 | 440.555g/mol | 90.743 | 3.5 | 3.503 |

**
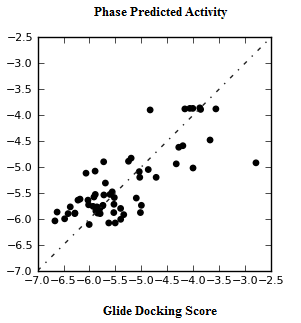
**

**FIGURE S1** Automatic generated regression plot of 3D QSAR with respect to Glide Docking Score and phase predicted activity, where X axis represents the Glide Docking Score and Y axis represents the phase predicted activity of chemical structure.

**
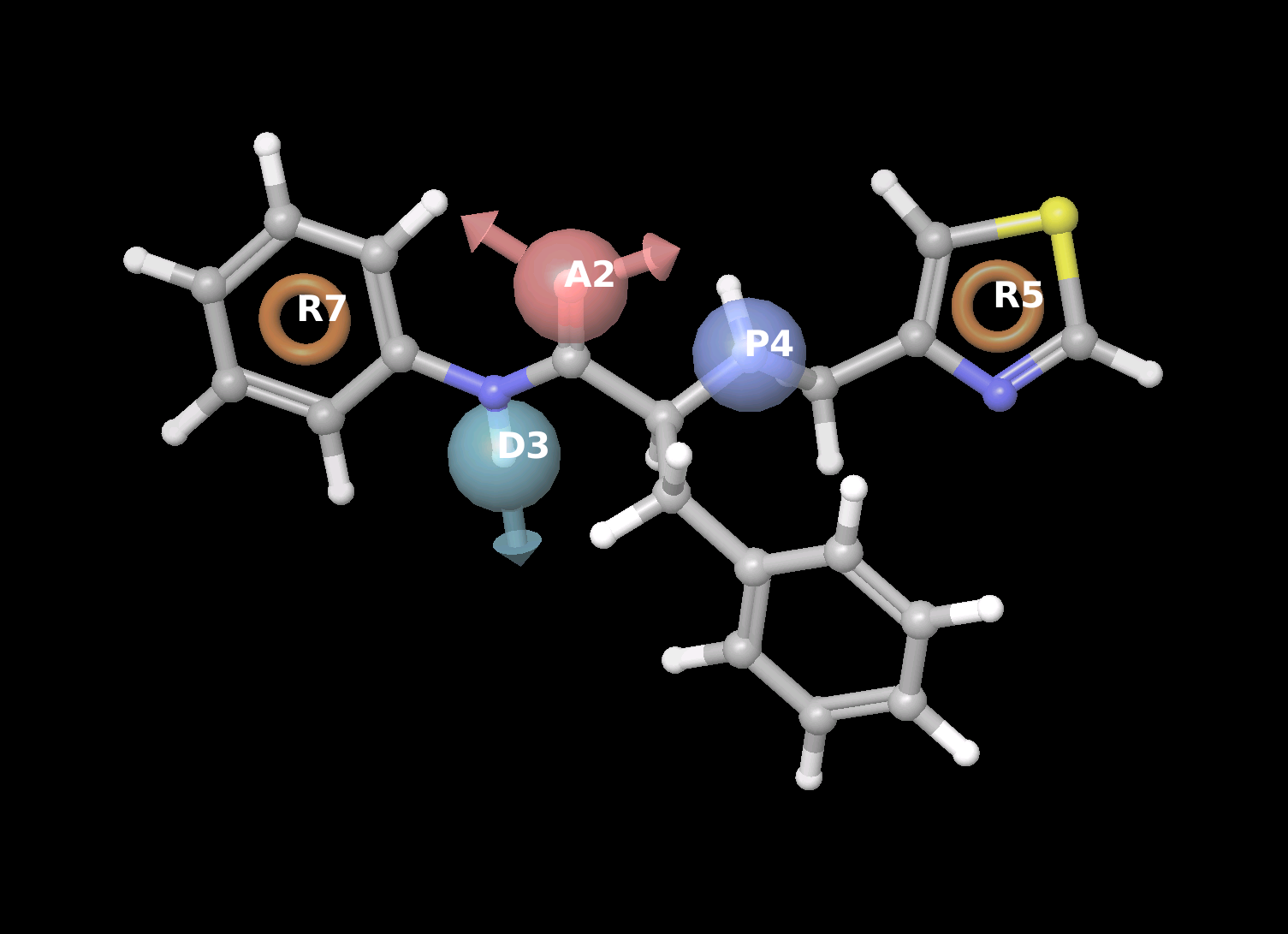
**

**FIGURE S2** Represents the common pharmacophore hypotheses, where R5 have most important common pharmacophoric feature that inhibit Type 2 diabetes.
